# The association between GP organisational factors and the effectiveness of a prevention programme for cardiometabolic diseases: a prospective intervention study

**DOI:** 10.3399/bjgpopen20X101111

**Published:** 2020-11-18

**Authors:** Ilse F Badenbroek, Daphne M Stol, Markus MJ Nielen, Monika Hollander, Niek J de Wit, François G Schellevis

**Affiliations:** 1 Julius Center For Health Sciences And Primary Care, UMC Utrecht, Utrecht, The Netherlands; 2 Netherlands Institute for Health Services Research (NIVEL), Utrecht, The Netherlands; 3 Department of General Practice & Elderly Care Medicine, Amsterdam Public Health Research Institute, Amsterdam University Medical Centers, Amsterdam, The Netherlands

**Keywords:** general practice, primary health care, primary prevention, cardiovascular diseases, risk assessment, organization and administration

## Abstract

**Background:**

Owing to the rising disease burden of cardiometabolic diseases (CMD), prevention programmes for CMD are increasingly implemented in primary care. Organisational practice characteristics and availability of preventive services may be associated with a more effective programme.

**Aim:**

To identify possible organisational success factors from general practices related to an effective primary prevention programme for CMD.

**Design & setting:**

A prospective intervention study involving 37 Dutch general practices was undertaken.

**Method:**

Patients aged 45–70 years without known CMD, hypertension, or hypercholesterolemia were invited for the prevention programme. The outcome measures were an improvement (yes/no) in four different CMD risk factors between baseline and 1-year follow-up on an individual level (body mass index [BMI], smoking, systolic blood pressure, and cholesterol ratio). Multivariate logistic regression analysis was used for assessing associations between practice organisational characteristics and outcomes.

**Results:**

Just over half of the participants showed an improvement on one or more risk factors. Marginal differences were found in the four different outcomes between the practices with different organisational characteristics. None of the practice characteristics that were tested showed a significant association with an improvement in one of the outcome measures.

**Conclusion:**

In this study, general practice organisational and preventive service characteristics showed no impact on the effectiveness of a CMD prevention programme. Possible explanations could be the effectiveness of protocolised pharmaceutical treatment and only limited contribution of lifestyle programmes on the improvement of CMD risk factors.

## How this fits in

It is known that organisational practice characteristics, such as practice type, support by non-medical staff, and an overview of available lifestyle services, are associated with improved quality indicators of standard cardiovascular prevention. But although prevention programmes for cardiometabolic diseases (CMD) are increasingly common in primary care, little is known about the relationship between practice organisational characteristics and the effectiveness of a selective CMD prevention programme. In this study it was found differences in general practice organisation and available preventive services in the practice had no impact on the effectiveness of a selective prevention programme for CMD. This might be explained by adequate protocolled pharmaceutical treatment and only a limited contribution of lifestyle programmes.

## Introduction

During the past decades healthcare systems have been confronted with an increasing disease burden of cardiometabolic diseases (CMD), including cardiovascular disease, type 2 diabetes mellitus, and chronic kidney disease. CMD are the number one cause of death globally and are accountable for more than half of all deaths across the World Health Organization European Region.^1^ Worldwide, an estimated 17.9 million people die of cardiovascular disease each year, diabetes causes another 1.6 million deaths yearly, and approximately 1.2 million people die from kidney failure.^1^ Lifestyle-related risk factors are accountable for 80% of all CMD.^2^ This has caused a shift from a curative to a more preventive approach, with counselling for a healthy lifestyle as an indispensable factor. Initiatives worldwide led to the development of different CMD prevention programmes,^3,4^ sharing the main goal to identify and treat people at high risk of CMD. Although previous studies have shown positive effects of prevention programmes for CMD in terms of risk profile improvement,^5,6^ evidence to support long-term effectiveness of these programmes is still missing.^3,4,7^

CMD prevention programmes are commonly organised within primary care. The GP is an easily accessible healthcare professional and, therefore, has a unique position within most healthcare systems to deliver a prevention programme. The GP is appointed as key-caregiver for CMD prevention in the most recent European guidelines on cardiovascular disease prevention in clinical practice.^2^ In everyday practice, however, preventive activities are often not prioritised by GPs.^8,9^ Improvements in practice organisation might help to overcome this paradox, for instance, a lack of time and focus can be tackled by deployment of practice nurses and lifestyle coaches, supporting the GP with preventive services. This leads to different methods of delivery of preventive programmes for CMD between practices, depending on available staff and other organisational practice characteristics.^9,10^ Earlier studies showed that organisational practice characteristics, such as practice type, support by non-medical staff, and an overview of available lifestyle services, are associated with improved quality indicators of standard cardiovascular prevention.^11–14^ Nevertheless, more than half of the general practices willing to participate in a selective CMD prevention programme fall short in offering adequate lifestyle support services and almost half of the practices lack an overview of available community-based lifestyle support services.^10^

Practice-related factors may be key in effective delivery of a CMD prevention programme, but little is currently known about this relationship. In order to address this gap in knowledge, the aim of this study was to identify whether organisational factors are related to the effectiveness of a CMD prevention programme in primary care.

## Method

### Design

This study is part of the INTEGRATE study, a Dutch stepped-wedge randomised controlled trial conducted from 2014–2017. A stepwise prevention programme for CMD^15^ followed by individualised treatment was implemented in 37 participating general practices. Details about the study design are described elsewhere,^16^ as well as the outcomes of the effectiveness of the prevention programme.^6^ The organisational characteristics of the 37 participating practices have been reported earlier.^10^

### Participants

All enlisted patients aged 45–70 years without known CMD, hypertension, or hypercholesterolemia, according to their electronic health record, were eligible for participation. Patients received a personal letter from their GP inviting them to complete the first step of the CMD prevention programme, the risk score. The risk score consisted of seven items, including sex, age, smoking status, BMI, waist circumference, and a family history of premature cardiovascular disease (age <65 years) and/or diabetes and resulted in the absolute risk to develop a CMD in the next 7 years.^17,18^ After filling in the risk score, online or on paper, participants with an increased risk for CMD (≥23% for men and ≥19% for women) were advised to visit the practice for the second step of the programme. At the practice, additional measurements were done, including blood pressure, cholesterol, and fasting glucose levels. During the third step of the programme, participants received tailored lifestyle advice and pharmaceutical treatment when indicated. All participants who filled in the online risk score received additional questionnaires.

For the present analysis, data were used from all participants who visited the general practice for additional profiling, confirmed in case report forms, electronic medical records, or by self-report. Missing baseline and outcome data on CMD risk factors were imputed using the multiple imputation techniques, described in more detail in the study describing the effectiveness of the programme.^6^

### Outcome variables

The primary outcome for this analysis was effectiveness of the CMD prevention programme, defined as an improvement in one or more CMD risk factors between baseline and 1-year follow-up on individual level. Individual CMD risk factors were smoking, systolic blood pressure, and total cholesterol/high density cholesterol ratio (TC/HDL ratio), all modifiable variables from the COronary Risk Evaluation (SCORE).^19^ BMI was added as an outcome variable for evaluation of lifestyle change, next to smoking status. Outcomes for BMI, systolic blood pressure, and TC/HDL ratio were dichotomised on an individual level into ‘no change or a deterioration (higher value)’ and ‘an improvement’ (that is, lower value) between baseline and follow-up. Data were collected from the electronic health record of the GP and through additional questionnaires.

### Practice characteristics

Questionnaires containing questions on the practice organisation and the delivery of CMD prevention were sent to all participating practices. The key professional in the implementation of the CMD prevention programme filled in the questionnaire. More details about the questionnaires and an overview of the characteristics of the participating practices at baseline is reported elsewhere.^10^

To prevent multiple testing a selection of characteristics with the highest potential was made, which was based on literature.^12–14^ The selected practice organisational characteristics were type of practice (single-handed, two GPs, or group practice of healthcare centre), practice setting (urban, urban–rural fringe, or rural), quality of care (practice accreditation and participation in chronic care group), health professionals in general practice (lifestyle coach and dietician), involvement in chronic disease management, lifestyle support service within general practice (weight management or healthy food sessions and exercise programmes), and community-based lifestyle services (informed about lifestyle services, written overview available, access to information during consultation).

### Analyses

Multivariate logistic regression analysis was used to assess the association between practice organisational characteristics and improvement in individual risk factors after 1-year follow-up. Outcomes were corrected for age and sex in all four different models. The analysis also corrected for clustering within practices. Odds ratios (ORs) and 95% confidential intervals (CIs) were used for reporting, all statistical analyses were performed using Stata (version 15.0).

## Results

Baseline organisational characteristics of the participating practices are shown in Table 1. A lifestyle coach was present in 16% of the participating practices, and weight and diet management or physical exercise programmes were offered in 30% and 14% of the practices, respectively. A total of 59% of the practices were well informed about available lifestyle programmes in the region.

**Table 1.** Baseline characteristics of participating general practices

**Practice characteristics (*n* = 37**)	**%**
**Type of practice (** **%)**	
Single-handed practice (1 GP)	27
Practice with 2 GPs	24
Group practice or healthcare centre (≥2 GPs)	49
**Practice setting (** **%)**	
Urban	46
Urban-rural fringe	16
Rural	38
**Quality of care (% yes** **)**	
Accreditation by NPA	73
Participation in chronic care group	89
**Health professionals in general practice (% yes** **)**	
Lifestyle coach	16
Dietician	51
**Involved in chronic disease management (% yes** **)**	
Cardiovascular risk management	82
**Lifestyle support service within general practice (% yes** **)**	
Weight management or healthy food sessions	30
Exercise programmes	14
**Community-based lifestyle services (% yes** **)**	
Practice is well informed about lifestyle services	59
Written overview of available lifestyle services	54
Access to information about lifestyle services during consultation	62

NPA = Netherlands Practice Accreditation.

From the 16 389 eligible individuals who were invited for the first step of the programme, 7313 (45%) completed the risk score; 2240 (31%) had an increased risk and were invited to contact their GP. A total of 967 participants (43% of those invited) visited the practice for additional profiling. An overview of the characteristics of the individual participants can be found elsewhere.^6^ Just more than half of the participants showed an improvement in BMI (52%), systolic blood pressure (51%), and TC/HDL ratio (53%) after 1 year of follow-up, and 4% of the smokers had stopped smoking.

Marginal differences were seen on the four different outcomes between practices with different organisational characteristics (Table 2). None of the practice characteristics analysed were significantly associated with outcome improvement. No clustering of outcome improvement was observed in any of the practice organisational characteristics, reaffirming that none of the characteristics were associated with an overall improvement in CMD-risk profile.

**Table 2.** Association between practice characteristics and percentage participants with improvement in CMD risk factors (adjusted ORs and 95% CI)

	**BMI**	**Smoking**	**Systolic blood pressure**	**TC/HDL ratio**
***n* =** **967**	% ↓^a^	OR [95% CI]	% ↓		OR [95% CI]	% ↓	OR [95% CI]	% ↓	OR [95% CI]
**Type of practice**									
Single-handed practice (1 GP) (reference)	48		9			54		58	
Practice with 2 GPs	50	1.10 [0.65 to 1.84]	3	0.31 [0.09 to 1.10]		47	0.78 [0.40 to 1.49]	48	0.65 [0.32 to 1.35]
Group practice or healthcare centre (≥2 GPs)	53	1.23 [0.80 to 1.91]	4	0.44 [0.18 to 1.07]		52	0.98 [0.56 to 1.70]	53	0.80 [0.43 to 1.46]
**Practice setting**									
Urban (reference)	53		3			51		52	
Urban–rural fringe	52	0.96 [0.65 to 1.41]	4	1.42 [0.49 to 4.08]		58	1.48 [0.86 to 2.56]	47	0.86 [0.49 to1.55]
Rural	50	0.90 [0.64 to 1.26]	6	2.25 [0.99 to 5.17]		48	0.92 [0.60 to 1.40]	59	1.31 [0.86 to 1.99]
**Quality of care**									
Accreditation by NPA (no/yes)	50/52	1.09 [0.78 to 1.53]	5/4	0.69 [0.30 to 1.59]		54/50	0.88 [0.55 to 1.42]	48/54	1.18 [0.71 to 1.96]
Participation in chronic care group (no/yes)	55/51	0.87 [0.60 to 1.25]	6/4	0.55 [0.24 to 1.29]		47/52	1.26 [0.74 to 2.14]	60/51	0.71 [0.41 to 1.21]
**Health professionals in general practice**									
Lifestyle coach (no/yes)	52/51	0.96 [0.67 to 1.37]	4/4	0.81 [0.30 to 2.24]		49/59	1.59 [1.00 to 2.52]	53/53	1.08 [0.64 to 1.82]
Dietician (no/yes)	49/53	1.19 [0.89 to 1.58]	5/4	0.70 [0.33 to 1.49]		47/54	1.36 [0.91 to 2.02]	59/49	0.70 [0.48 to 1.04]
**Involved in chronic disease management**									
Cardiovascular risk management (no/yes)	49/53	1.15 [0.80 to 1.67]	5/4	0.94 [0.38 to 2.38]		49/52	1.10 [0.68 to 1.78]	54/53	0.97 [0.60 to 1.57]
**Lifestyle support service within general practice**									
Weight management or healthy food sessions (no/yes)	51/54	1.15 [0.84 to 1.56]	4/4	0.98 [0.44 to 2.20]		49/55	1.32 [0.87 to 2.01]	53/52	0.97 [0.60 to 1.57]
Exercise programmes (no/yes)	52/47	0.80 [0.43 to 1.49]	4/0	1.00 [1.00 to 1.00]		51/62	1.65 [0.79 to 3.43]	53/54	1.09 [0.47 to 2.54]
**Community-based lifestyle services**									
Practice is well informed about lifestyle services (no/yes)	51/53	1.08 [0.81 to 1.45]	4/5	1.30 [0.61 to 2.79]		51/51	1.02 [0.67 to 1.57]	54/52	1.05 [0.66 to 1.66]
Written overview of available lifestyle services (no/yes)	51/54	1.13 [0.85 to 1.49]	4/4	1.13 [0.52 to 2.44]		52/51	0.94 [0.62 to 1.42]	51/55	1.30 [0.83 to 2.02]
Information lifestyle services during consultation (no/yes)	50/53	1.12 [0.84 to 1.50]	4/4	1.14 [0.53 to 2.48]		54/49	0.77 [0.52 to 1.15]	52/54	1.09 [0.79 to 1.52]

ORs adjusted for age and sex at individual level and adjusted for clustering at practice level. CI = confidence intervals. OR = odds ratio. NPA = Netherlands Practice Accreditation.

a Percentage of participants who showed an improvement in CMD risk factor (for example, lower BMI).

BMI body mass index OR odds ratio CMD cardiometabolic diseases CI confidence interval TC/HDL ratio TC/HDL ratio = total cholesterol/high density cholesterol ratio

## Discussion

### Summary

This study aimed to identify organisational characteristics of primary care practices that were associated with the effectiveness of a prevention programme for CMD. Although all four individual CMD risk factors improved for the majority of patients, none of the practice characteristics were significantly associated with this improvement. Based on the data, practice organisation does not seem to contribute to the effectiveness of CMD prevention programmes in general practice.

### Strengths and limitations

This study was part of a large randomised controlled trial with a pragmatic approach, making the results representative for a ‘real-life setting’ in primary care. Another strength was the use of actual change in risk factors for CMD on an individual level, in contrast to earlier studies using indicators of performance (for example, percentage of recorded risk factors or percentage of patients with achieved protocolled treatment targets) derived from electronic health records as a measure for the quality of preventive care delivery. The total number of general practices used in the analysis was small compared with earlier studies that assessed practice characteristics.^11–14^ On the other hand, with both rural and urban practices of variable sizes, the study practices were heterogeneous enough to be representative for Dutch general practice and their patient population.^6^ The available data on individual level was limited to the 976 participants that finished step two of the prevention programme, divided between the 37 practices. A larger dataset would have increased the validity of the results. The final limitation of this study is the generalisability of the results. The extent to which the results can be extrapolated to other countries might be limited, for healthcare systems might not be comparable, and it is unclear how the organisational practice factors of Dutch general practices relates to practices in other countries.

### Comparison with existing literature

To the author's knowledge, this is the first study to investigate the relationship between practice organisational characteristics and the effectiveness of a prevention programme for CMD. The study results do not compare well with the outcomes of earlier research because of crucial differences in study aim and design. Earlier research focused mainly on the association between practices' characteristics and the quality of standard cardiovascular management for patients with mostly known cardiovascular disease. In these earlier studies, practices' characteristics were associated with a better performance in some process quality indicators for standard cardiovascular prevention.^11–14^ Nevertheless, none of these practice characteristics were associated with an improvement in CMD risk factor outcome in newly detected high-risk patients after 1-year follow-up in the study.

Even though practices vary in organisational factors and availability of preventive services, pharmaceutical treatment protocols for individuals are standardised in The Netherlands. Practices with a lifestyle coach, dietician, or lifestyle support services do not have better outcomes than practices without these facilities. This suggests a lack in effectiveness of offering lifestyle programmes for this population, either by too few referrals, a low attendance rate, or low effectiveness of the lifestyle programmes themselves. Lifestyle changes probably only have a limited additional contribution to the effect of antihypertensive and anti-hypercholesterolemia treatment,^6^ which explains the small differences in outcomes found in the present study.

### Implications for research and practice

In the INTEGRATE study, differences in general practice organisational characteristics and availability of preventive services showed no impact on the effectiveness of a CMD prevention programme, possibly owing to the highly standardised pharmaceutical treatment and the limited contribution of lifestyle programmes to CMD risk factor improvement. These exploratory findings should be viewed in the light of sample size limitations and further research to confirm these findings is warranted. Future research should also focus on the development of effective lifestyle programmes before valid recommendations about the organisation of preventive services for primary prevention of CMD in the general practice can be made.

## Trial registration number

Dutch Trial Register Number NTR4277 for the INTEGRATE study.
